# Ce-Duox1/BLI-3 Generated Reactive Oxygen Species Trigger Protective SKN-1 Activity via p38 MAPK Signaling during Infection in *C. elegans*

**DOI:** 10.1371/journal.ppat.1002453

**Published:** 2011-12-22

**Authors:** Ransome van der Hoeven, Katie C. McCallum, Melissa R. Cruz, Danielle A. Garsin

**Affiliations:** Department of Microbiology and Molecular Genetics, The University of Texas Health Science Center at Houston, Houston, Texas, United States of America; Massachusetts General Hospital, Harvard Medical School, United States of America

## Abstract

Infected animals will produce reactive oxygen species (ROS) and other inflammatory molecules that help fight pathogens, but can inadvertently damage host tissue. Therefore specific responses, which protect and repair against the collateral damage caused by the immune response, are critical for successfully surviving pathogen attack. We previously demonstrated that ROS are generated during infection in the model host *Caenorhabditis elegans* by the dual oxidase Ce-Duox1/BLI-3. Herein, an important connection between ROS generation by Ce-Duox1/BLI-3 and upregulation of a protective transcriptional response by SKN-1 is established in the context of infection. SKN-1 is an ortholog of the mammalian Nrf transcription factors and has previously been documented to promote survival, following oxidative stress, by upregulating genes involved in the detoxification of ROS and other reactive compounds. Using qRT-PCR, transcriptional reporter fusions, and a translational fusion, SKN-1 is shown to become highly active in the *C. elegans* intestine upon exposure to the human bacterial pathogens, *Enterococcus faecalis* and *Pseudomonas aeruginosa.* Activation is dependent on the overall pathogenicity of the bacterium, demonstrated by a weakened response observed in attenuated mutants of these pathogens. Previous work demonstrated a role for p38 MAPK signaling both in pathogen resistance and in activating SKN-1 upon exposure to chemically induced oxidative stress. We show that NSY-1, SEK-1 and PMK-1 are also required for SKN-1 activity during infection. Evidence is also presented that the ROS produced by Ce-Duox1/BLI-3 is the source of SKN-1 activation via p38 MAPK signaling during infection. Finally, for the first time, SKN-1 activity is shown to be protective during infection; loss of *skn-1* decreases resistance, whereas increasing SKN-1 activity augments resistance to pathogen. Overall, a model is presented in which ROS generation by Ce-Duox1/BLI-3 activates a protective SKN-1 response via p38 MAPK signaling.

## Author Summary

To fight infection, an animal's immune response produces a variety of toxic compounds that are directed at the invading pathogen. However, these toxic compounds can also damage the host's tissue. Therefore an important job of the immune response is to prevent and repair damage caused by “friendly fire.” In this study, we explore this damage control function of the immune response in a small worm called *C. elegans*, which can be infected by feeding on human pathogens and serves as a model of human infection. Upon exposure of *C. elegans* to reactive compounds, a factor called SKN-1 was shown to induce the production of protective, detoxifying enzymes. Here, we demonstrate that SKN-1 also becomes active in infected animals. The cause of SKN-1 activation is reactive compounds produced by the animal's immune system, i.e. a source of “friendly fire.” We additionally show that a highly conserved signaling pathway, called the p38 MAPK pathway, controls SKN-1 activity during infection. Finally, we demonstrate that SKN-1 activity is beneficial to the worm during infection, enhancing survival. Because humans share many of the components of the interaction we describe, this study provides broad insights into the principals of damage control during immune response.

## Introduction

Infection by pathogenic microorganisms requires a coordinated response from the host to cope with the multitude of physiological challenges presented by the attack. In addition to producing compounds that have direct antimicrobial activity and countering pathogen virulence strategies, the host must also initiate stress responses to protect cellular resources and processes from the negative consequences of “friendly-fire.” Damage, disease, and sometimes death of the host can occur if immune responses are not controlled or protected against properly. Septic shock and various autoimmune diseases are examples of immune responses gone awry. In this work we explore the connections between infection, immune response and cellular stress response using the well-studied model host *Caenorhabditis elegans.*

A general response to microbial challenge that most animals possess is the production of reactive oxygen species (ROS). The best-studied example is the production of ROS as an antimicrobial response in the phagolysosome of phagocytic cells by the NADPH oxidase gp91*^phox^*. However, this response is not limited to phagocytes, and NADPH oxidases are present in the skin as well as the mucosal epithelium of the oral cavity, respiratory and gastrointestinal tracts of humans [1], [2]. Less complex organisms that lack innate immune cells, such as *C. elegans*, also encode for NADPH oxidases. For instance, the dual oxidase Ce-Duox1/BLI-3 is present in the hypodermis and in the intestine of *C. elegans* [3], [4]. Our laboratory and others recently demonstrated that an intestinal infection triggers the release of ROS by Ce-Duox1/BLI-3 in what appears to be a protective response [3], [5]. Presumably due to the production of ROS however, there was also evidence of cellular damage as shown by lipofuscin accumulation and loss of protein homeostasis, which was worsened by the knock-down of certain oxidative stress enzymes [6], [7]. The goal of this work was to determine if infection, by triggering ROS release by Ce-Duox1/BLI-3, induces an oxidative stress response in the host as part of the overall response to the pathogen.

SKN-1 is a transcription factor that senses oxidative stress and functionally affects resistance to oxidative stress and lifespan in *C. elegans*. It is an Nrf ortholog, a protein family found in all eukaryotes that upregulates the Phase 2 genes of the three-phase detoxification system [8]. Phase 2 genes encode enzymes that defend against ROS and other reactive compounds [9]. A large number of genes are regulated by SKN-1, including many glutathione-S-transferases that are important for detoxifying reactive compounds such as xenobiotics and peroxidized lipids by conjugating glutathione to electrophilic centers [10], [11].

SKN-1 transcriptional activity is regulated by phosphorylation by the p38 MAPK, PMK-1, which promotes its localization to the nucleus [12]. Interestingly, PMK-1 is a major regulator of *C. elegans* innate immunity and loss of this protein results in a strong susceptibility phenotype [13]. Work on PMK-1 showed that it is regulated by a phosphorylation cascade involving the activation of a Toll/IL-1 receptor (TIR) domain protein, TIR-1, which leads to the activation of a MAPKKK called NSY-1, which then activates a MAPKK called SEK-1, culminating in PMK-1 phosphorylation [13], [14]. Phosphorylation of the transcription factor ATF-7 by PMK-1 is thought to ultimately promote innate immune gene expression by turning this repressor of innate immune gene expression into an activator [15]. While SEK-1 is also required for activation of SKN-1 by oxidative stress [12], the roles of TIR-1 and NSY-1 are controversial. One report shows that NSY-1 and TIR-1 are dispensable for activation of PMK-1 in response to oxidative stress created by sodium arsenite [12], whereas two other publications show that NSY-1 is required for resistance to oxidative stress caused by paraquat [16]–[18].

In earlier studies, loss of SKN-1 was not observed to affect the overall susceptibility of the worm to infection by *Pseudomonas aeruginosa*, and it was postulated that the oxidative stress transcriptional response mediated by SKN-1 is not involved in pathogen defense [15], [19]. However, because our data suggested that oxidative stress is present during infection [3], [6], [7], we postulated that SKN-1 is activated and conducted experiments to investigate this hypothesis.

Specifically, this study examined SKN-1 directed gene expression and localization in *C. elegans* infected with *Pseudomonas aeruginosa* and *Enterococcus faecalis*. We establish that bacterial infection stimulates SKN-1 activity in a manner dependent on the p38 MAPK signaling pathway. We show that components of the p38 MAPK signaling pathway previously established as necessary for responding to pathogen are involved, including NSY-1, SEK-1 and PMK-1, but not TIR-1. In contrast to previous work, we find that loss of SKN-1 activity increases susceptibility to the pathogens whereas constitutive activation results in increased resistance. Of key significance is the demonstration that ROS produced by Ce-Duox1/BLI-3 is the source of oxidative stress triggering SKN-1 activity during infection. Overall, this work establishes that a protective SKN-1 response is activated during infection via p38 MAPK signaling as a result of the mucosal oxidative burst generated by the host.

## Results

### Pathogen Infection Induces Expression of SKN-1 Controlled Genes

**Figure 1:**
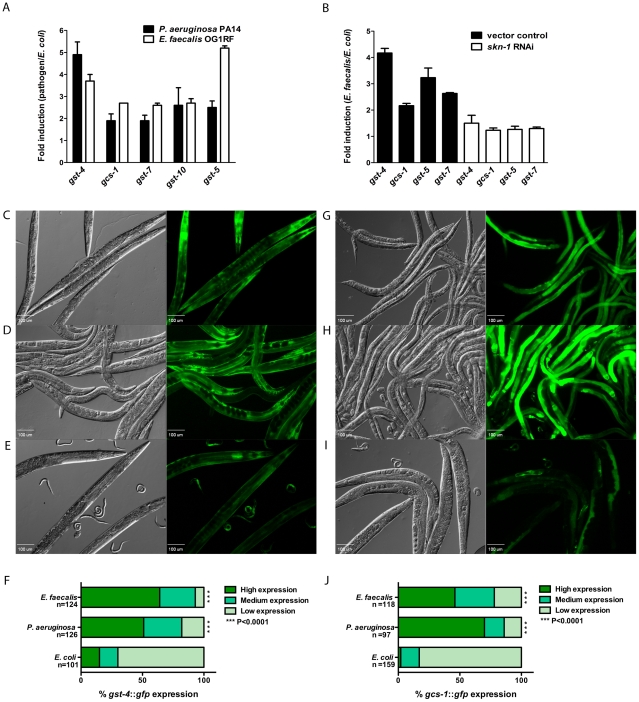
SKN-1 dependent genes are activated in response to pathogens. (**A & B**) qRT-PCR analysis of SKN-1 dependent genes induced in worms fed for 24 hours on *E. faecalis* OG1RF, *P. aeruginosa* PA14 and *E. coli* OP50, and in *skn-1* and control RNAi worms fed on *E. faecalis* OG1RF and *E. coli* OP50. Experiments were performed with three separate replicates; each replicate was measured in duplicate and normalized to the control gene *act-1*. Error bars represent the standard error of the mean (SEM). (**C - E**) The expression pattern of *gst-4::gfp* in worms exposed to *E. faecalis* OG1RF, *P. aeruginosa* PA14 and *E. coli* OP50 for 18 hours. (**F**) The level of *gst-4::gfp* expression was scored as described in Materials and Methods and the percentage of worms in each category is indicated along with the number of worms observed (n). (**G - I**) The expression pattern of *gcs-1::gfp* in worms exposed to *E. faecalis* OG1RF, *P. aeruginosa* PA14 and *E. coli* OP50 for 18 hours. (**J**) The level of *gcs-1::gfp* expression was scored as described in Materials and Methods and the percentage of worms in each category is indicated along with the number of worms observed (n). In the micrographs, Normaski and fluorescent views of the worms are depicted. *E. faecalis* OG1RF and *P. aeruginosa* PA14 induced significantly higher levels of *gst-4::gfp* and *gcs-1::gfp* (*P*<0.0001) compared to *E. coli* OP50.

Previous work in our laboratory demonstrated that *C. elegans* releases significant amounts of ROS during infection and that expression of oxidative stress response genes such as *sod-3* is induced [6]. Additionally, we have shown that cells at the site of the infection, the intestine, display a loss of protein homeostasis indicative of cellular stress [7]. SKN-1 is a transcription factor that responds to cellular stressors, including ROS [8], and we predicted that its activity is likely to be induced as a result of pathogen exposure. We tested genes previously characterized as being regulated by SKN-1, including *gst-4, gst-5, gst-7, gst-10* and *gcs-1*. The *gst* genes encode for glutathione-S-transferases, which are important for detoxifying reactive compounds by conjugating reduced glutathione to electrophilic centers, while *gcs-1* encodes for gamma-glutamine cysteine synthetase heavy chain, a protein involved in glutathione biosynthesis [10], [11], [20]. Expression of these genes was examined by qRT-PCR following a 24-hour exposure of L4 worms to both *P. aeruginosa* and *E. faecalis* (Figure 1A). All of the reporter genes were induced between two and five-fold more when the animals were feeding on the pathogens, as compared to those feeding on their normal laboratory food source (*E. coli* OP50). As shown in Figure 1B, in which exposure of the animals to *skn-1* RNAi preceded exposure to *E. faecalis*, the induction was prevented, indicating that expression of these genes retains dependence on SKN-1 under conditions of pathogen exposure.

The Blackwell and Johnson laboratories have generated several *C. elegans* strains containing GFP reporter fusions to genes that are markers for SKN-1 activity including *gst-4* [21], *gcs-1* [8], [22] and *gst-7* [20]. We obtained these strains to further examine pathogen-induced SKN-1 activity. L4 animals were exposed to *P. aeruginosa, E. faecalis* or non-pathogenic *E. coli* for 18 hours. Fluorescent micrographs were taken, and the animals were scored for low, medium or high expression of the reporters as described in Methods. For each condition, several worms in a representative micrograph are shown (Figures 1C-E, 1G-I), and additionally the quantification of the scoring with statistical analysis is included (Figures 1F and 1J). Figures 1C-F show that significantly more animals had high levels of expression of *gst-4::gfp* when infected with *E. faecalis* (Figure 1C) and *P. aeruginosa* (Figure 1D) than when allowed to feed on *E. coli* (Figure 1E). Significantly higher expression of *gcs-1::gfp* was also observed on the pathogenic strains compared to the controls as shown in Figures 1G-J. We also examined the levels of GST-7::GFP using a transgenic strain containing a translational fusion of *gfp* to the *gst-7* gene, and again we observed higher levels when the animals were exposed to the pathogens (Figure S1 in Text S1). To ensure that the expression was SKN-1 dependent, the levels of fluorescence were observed in strains exposed to *skn-1* RNAi prior to pathogen exposure; the pathogen-induced increase in fluorescence was abolished, supporting the use of these reporters as read-outs for SKN-1 expression levels (see below, S2E in Text S1 and data not shown). In independent experiments looking at number of transcripts by RNAseq experiments that examine transcript numbers in worms exposed to another human pathogen, *Staphylococcus aureus,* both *gcs-1* and *gst-4* were expressed at higher levels compared to animals on *E. coli* (Javier Irazoqui, personal communication, data not shown). In conclusion, several different experimental methodologies indicate that SKN-1 regulated genes have increased levels of expression in *C. elegans* fed on pathogenic bacteria.

### Attenuated Pathogen Mutants Reduce Induction of SKN-1 Controlled Genes

**Figure 2:**
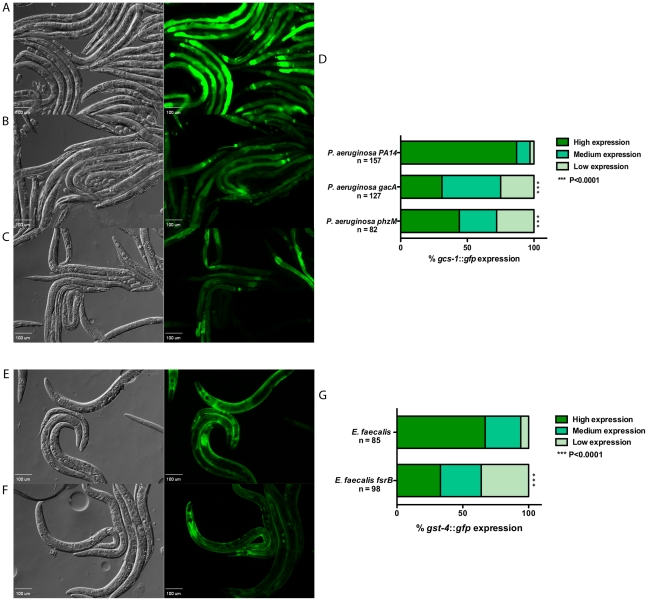
Expression of SKN-1 regulated genes is reduced in response to attenuated mutants of *P. aeruginosa* PA14 and *E. faecalis* OG1RF. (**A – C**) The expression of *gcs-1::gfp* in worms fed on wild-type *P. aeruginosa* PA14, and mutant strains *gacA* and *phzM* for 18 hours. (**D**) The level of *gcs-1::gfp* expression was scored and the percentage of worms in each category is indicated along with the number of worms observed (n). (**E & F**) The expression of *gst-4::gfp* in worms fed on wild-type *E. faecalis* OG1RF and an *fsrB* mutant strain for 18 hours. (**G**) The level of *gst-4::gfp* expression was scored and the percentage of worms in each category is indicated along with the number of worms observed (n). In the micrographs, Normaski and fluorescent views of the worms are depicted. Wild-type strains induced significantly higher levels of *gst-4::gfp* and *gcs-1::gfp* expression (*P*<0.0001) compared to mutant strains of the pathogens.

To investigate whether or not the strength of the SKN-1 response is affected by any known bacterial virulence factors, we tested some well-studied mutants. GacA is a response regulator in *P. aeruginosa* that strongly affects the virulence of this pathogen and a *gacA* mutant is greatly attenuated in a variety of hosts including *C. elegans* [23]. As shown in Figure 2B, a *gacA* deletion mutant significantly reduced the expression level of the *gcs-1::gfp* reporter as compared to the isogenic parental strain of *P. aeruginosa* shown in Figure 2A. One of the GacA-dependent virulence factors is pyocyanin [24], a secreted compound that is redox active and has been shown to oxidatively stress host cells [25]–[27]. We examined a pyocyanin-deficient mutant, which is deleted in *phzM,* a gene that encodes an enzyme critical for pyocyanin biosynthesis [28], [29]. As seen in Figure 2C, this mutant also results in less *gcs-1::gfp* reporter expression as compared to wild-type. The quantification of the effects of these *P. aeruginosa* mutants on *gcs-1::gfp* expression is shown in Figure 2D. In *E. faecalis*, FsrB is a component of a quorum sensing system that has been found to affect virulence in every animal model studied, including *C. elegans* [30], [31]. An *fsrB* deletion mutant also resulted in less SKN-1 activity, as measured by *gst-4::gfp* expression compared to the isogenic parental strain (Figure 2E-G). These results show that the level of SKN-1 activity is sensitive to the presence or absence of major virulence factors and that SKN-1 activity may be a way to discern the overall virulence of the infecting organism.

### Pathogen Exposure Induces SKN-1 Nuclear Localization

**Figure 3:**
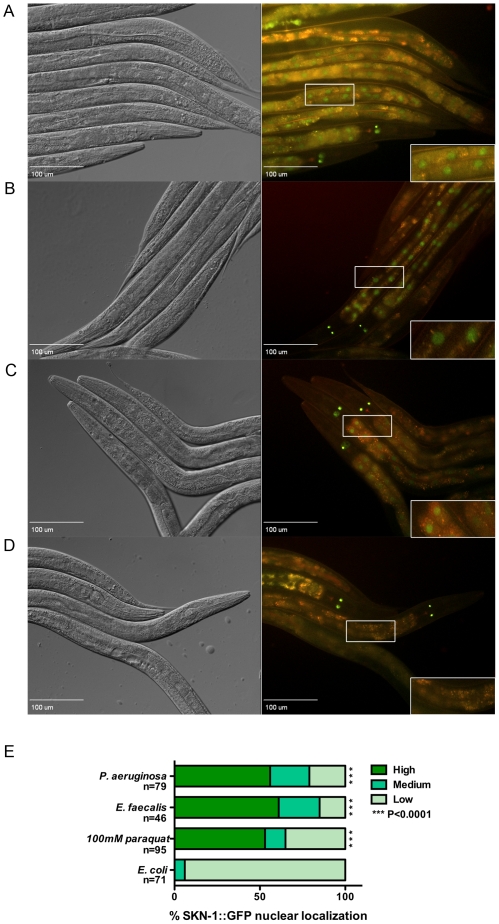
SKN-1 is localized to the nuclei of the intestinal cells in response to the pathogens. Worms integrated with SKN-1B/C::GFP transgene were exposed to (**A**) *P. aeruginosa* PA14 for 6 hrs, (**B**) *E. faecalis* OG1RF for 24 hrs, (**C**) 100 mM paraquat for 30 minutes and (**D**) *E. coli* OP50 for 24 hrs. SKN-1B/C::GFP localization was observed by fluorescence microscopy. Close-ups of the boxed region are shown in the lower right-hand corner of each fluorescent image. (**E**) The degree of nuclear localization was scored as described in Materials and Methods and is given as a percentage for each category. The number of worms used in scoring each experimental condition is indicated (n). Significantly higher nuclear localization of SKN-1B/C::GFP was observed in worms exposed to the pathogens and paraquat (*P*<0.0001) compared to *E. coli* OP50.

SKN-1 activity is regulated by localization to the nucleus [8], [32]. If pathogen exposure increases SKN-1 activity, one would expect to see localization of this transcription factor to the nucleus. We obtained a SKN-1B/C::GFP transgenic line used in previous studies to examine SKN-1 activity and localization [8]. As shown in Figure 3, exposure to *P. aeruginosa* (Figure 3A) or *E. faecalis* (Figure 3B) causes significant nuclear localization of SKN-1B/C::GFP, similar to what is observed by exposure to paraquat (Figure 3C), a chemical that causes oxidative stress and is documented to promote SKN-1 nuclear localization [8]. In contrast, when feeding on their normal laboratory food source, the animals do not display significant SKN-1B/C::GFP nuclear localization (Figure 3D).

### Pathogen-Induced Expression of SKN-1 Genes Is Dependent on p38 MAPK Signaling

**Figure 4:**
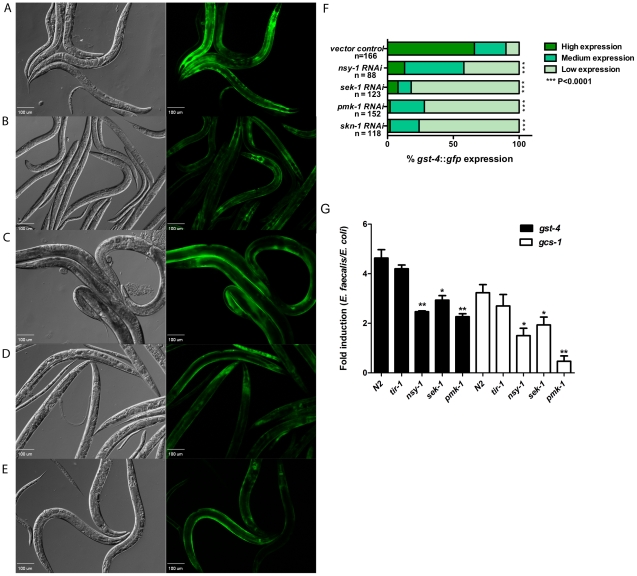
Activation of SKN-1 in response to pathogens is dependent on the p38-MAPK pathway. Representative Nomarski and fluorescent views of worms expressing *gst-4::gfp* exposed to (**A**) control, (**B**) *nsy-1*, (**C**) *sek-1*, (**D**) *pmk-1*, and (**E**) *skn-1* RNAi prior to feeding *E. faecalis* OG1RF for 18 hours. (**F**) The level of *gst-4::gfp* expression was scored and the percentage of worms in each category is indicated along with the number of worms observed (n). Significantly higher levels of expression of *gst-4::gfp* were observed in worms exposed to the vector control RNAi (*P*<0.0001) as compared to *nsy-1*, *sek-1*, *pmk-1* and *skn-1* RNAi. (**G**) qRT-PCR analysis of SKN-1 dependent genes *gst-4* and *gcs-1* induced in N2, *tir-1(qd4)III, nsy-1(ag3)II, sek-1(ag1)X* and *pmk-1*(*km25*)IV worms exposed to *E. faecalis* OG1RF and *E. coli* OP50 for 24 hours. Significantly lower levels of *gst-4* and *gcs-1* gene expression were observed in the *nsy-1(ag3)II*, *sek-1(ag1)* and *pmk-1(km25)* compared to the N2 strain. * *P*<0.01 and ** *P*<0.001 respectively.

Utilizing chemicals known to generate oxidative stress (paraquat, sodium arsenite and t-butyl peroxide), previous work demonstrated that activation of SKN-1 is dependent on p38 MAPK signaling components [12], [16], [17]. We asked whether or not pathogen-induced activation of SKN-1 is also dependent on p38 MAPK signaling. To investigate this question, the levels of GFP were scored in the *gst-4::gfp* transgenic worms after RNAi knock-down of the genes-of-interest followed by 18 hours of exposure to *E. faecalis* (Figure 4). Additionally, we assayed *gcs-1::gfp* transgenics exposed to *P. aeruginosa* following RNAi (Figure S2 in Text S1). As a control, the effects of RNAi knock-down on animals exposed to the non-pathogenic control, *E. coli*, were also assayed and found to be minimal (Table S1 in Text S1).

The p38 MAPK, PMK-1, and the upstream MAPKK, SEK-1, are absolutely necessary to activate SKN-1 as a result of oxidative stress [12]. As shown in Figure 4, reduction in the expression of *sek-1* (Figure 4C) or *pmk-1* (Figure 4D) by RNAi prior to exposure to *E. faecalis* resulted in significantly less fluorescence of the SKN-1 dependent reporter, *gst-4::gfp*, in the worm intestine, with similar levels to the *skn-1* RNAi control (Figure 4E). These data suggest that PMK-1 and SEK-1 are also crucial for SKN-1 activation as a result of pathogen exposure. RNAi of these genes had similar effects on *gcs-1::gfp* transgenics exposed to *P. aeruginosa* (Figure S2C and S2D in Text S1).

In addition to its role in responding to oxidative stress, it was previously demonstrated that PMK-1 is activated by pathogen exposure and plays a very important role in host defense. The infection protective activity of PMK-1 is dependent not only on SEK-1, but also on the upstream MAPKKK NSY-1 and the Toll/IL-1 receptor (TIR) domain protein, TIR-1 [13], [14]. The role of NSY-1 and TIR-1 in PMK-1 activation under conditions of oxidative stress is less clear and may be dependent on the stressor utilized [12], [16], [17]. To investigate the possible roles of NSY-1 and TIR-1 on SKN-1 activation during pathogen exposure, we exposed the animals to *nsy-1* or *tir-1* RNAi prior to placing them on pathogen. Knock-down of *nsy-1* caused loss of SKN-1 activity on both pathogens as assayed by the *gst-4::gfp* and *gcs-1::gfp* reporters, (Figure 4B, Figure S2B in Text S1). In contrast, knock-down of *tir-1,* resulted in only a non-statistically significant trend towards less expression, suggesting no, to minimal involvement, at best (Figure S3B in Text S1 and data not shown).

To confirm the RNAi results using another methodology, we also examined expression in genetic mutants using qRT-PCR to measure the expression of *gst-4* and *gcs-1*. In null mutants of *nsy-1*, *sek-1* and *pmk-1*, expression of these two genes was down relative to wild-type worms on both *E. faecalis* and *P. aeruginosa* (Figure 4G). In contrast, expression levels were not significantly different in a *tir-1* mutant.

### Pathogen-Induced Expression of SKN-1 Genes Is Dependent on Ce-Duox1/BLI-3

**Figure 5:**
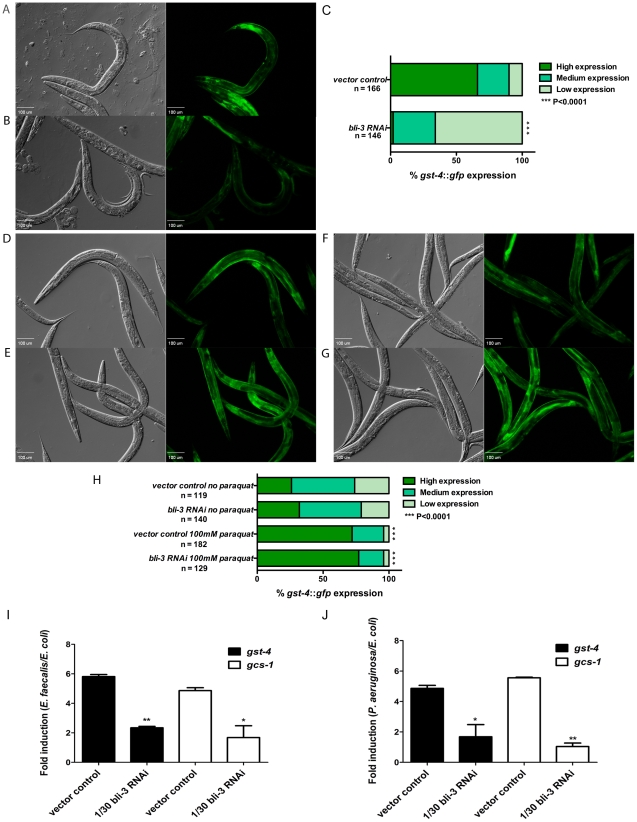
SKN-1 dependent genes are induced in response to ROS produced by Ce-Duox1/BLI-3 during infection. Expression of *gst-4::gfp* in worms exposed to vector control (**A**) and 1/30 *bli-3* (**B**) RNAi prior to feeding on *E. faecalis* OG1RF for 18 hours. Representative Normaski and fluorescent views of the worms are depicted. (**C**) The level of *gst-4::gfp* expression was scored and the percentage of worms in each category is indicated along with the number of worms observed (n). Significantly higher levels of expression of *gst-4::gfp* were observed in worms exposed to the control (*P*<0.0001) compared to 1/30 *bli-3* RNAi prior to exposure to the pathogen. Images of vector control (**D** and **E**) and 1/30 *bli-3* (**F** and **G**) RNAi worms expressing *gst-4*::*gfp* in the presence of M9 (**D** and **F**) and paraquat (**E** and **G**) for 30 minutes. (**H**) The level of *gst-4::gfp* expression was scored and the percentage of worms in each category is indicated along with the number of worms observed (n). No significant difference in expression was observed between the control and the 1/30 *bli-3* RNAi worms when exposed to paraquat (*P* = 0.5694). Similar results were obtained in three independent replicates of each experiment. qRT-PCR analysis of SKN-1 dependent genes *gst-4* and *gcs-1* induced in 1/30 *bli-3* RNAi and control worms when exposed to *E. faecalis* OG1RF (**I**) and *P. aeruginosa* PA14 (**J)** compared to *E. coli* OP50 for 6 hours. Significantly lower levels of *gst-4* and *gcs-1* expression were observed in 1/30 *bli-3* knockdown animals on both pathogens compared to the control. * *P*<0.01 and ** *P*<0.001 respectively.

Our previous studies demonstrated that part of the response of the worm to pathogen challenge is the release of ROS into the intestinal lumen by the dual oxidase Ce-Duox1/BLI-3. Rather than a tangential consequence of cell death, this response is purposeful and protective [3], [6]. Since SKN-1 is known to respond to oxidative stress, and the above-described experiments conducted on the p38 MAPK signaling pathway are most consistent with an oxidative stress response, we postulated that the ROS generated by Ce-Duox1/BLI-3 during pathogen exposure may trigger signaling through the p38 MAPK pathway, resulting in SKN-1 activation. To test this, we knocked down the expression of *bli-3* by RNAi in the *gst-4::gfp* background and then exposed the strain to *E. faecalis.* As shown in Figure 5A-C, SKN-1 activity is reduced to a level comparable to worms exposed to *skn-1* RNAi (Figure 4E), suggesting that the presence of Ce-Duox1/BLI-3 is necessary for SKN-1 activity. We propose that the ROS produced by Ce-Duox1/BLI-3 in response to pathogens and the resulting oxidative stress activates SKN-1 activity.

To test if SKN-1 activity could be rescued in the *bli-3* knock-down worms by providing ROS from an alternative source, we exposed the animals to paraquat. Paraquat generates ROS by redox cycling in vivo and has been used in previous studies as a trigger for SKN-1 activity in *C. elegans* [8]. L4 worms were exposed to either 1/30 *bli-3* RNAi or vector control RNAi and were placed for 30 minutes in M9 solution with or without 100 mM of paraquat. As shown in Figure 5E, SKN-1 activity, as measured by fluorescence of the *gst-4::gfp* fusion, was activated in response to paraquat in worms fed vector control RNAi, in agreement with previous work [8]. Knock-down of *bli-3* had no effect on activation of SKN-1 by paraquat (Figure 5G). These data indicate that SKN-1 activation induced by a chemical ROS generator does not require Ce-Duox1/BLI-3, unlike activation by pathogen exposure.

We were unable to perform these experiments on *P. aeruginosa* using the *gfp-*expressing transgenics because we discovered that the *bli-3* knock-down caused a severe susceptibility phenotype on *P. aeruginosa*, much more severe than on *E. faecalis*, and more than half the worms were dead by the 24 hour time point (see below). Instead we used qRT-PCR to look at SKN-1 regulated genes on both *E. faecalis* and *P. aeruginosa* at an earlier time point (6 hours). By this methodology, we observed that both *gst-4* and *gcs-1* expression was significantly reduced in the *bli-3* knock-downs compared to wild-type on both pathogens (Figure 5I and 5J). In conclusion, our results support a model in which ROS generated by Ce-Duox1/BLI-3 as a result of pathogen exposure trigger SKN-1 activity.

### SKN-1 Influences Pathogen Susceptibility

**Figure 6:**
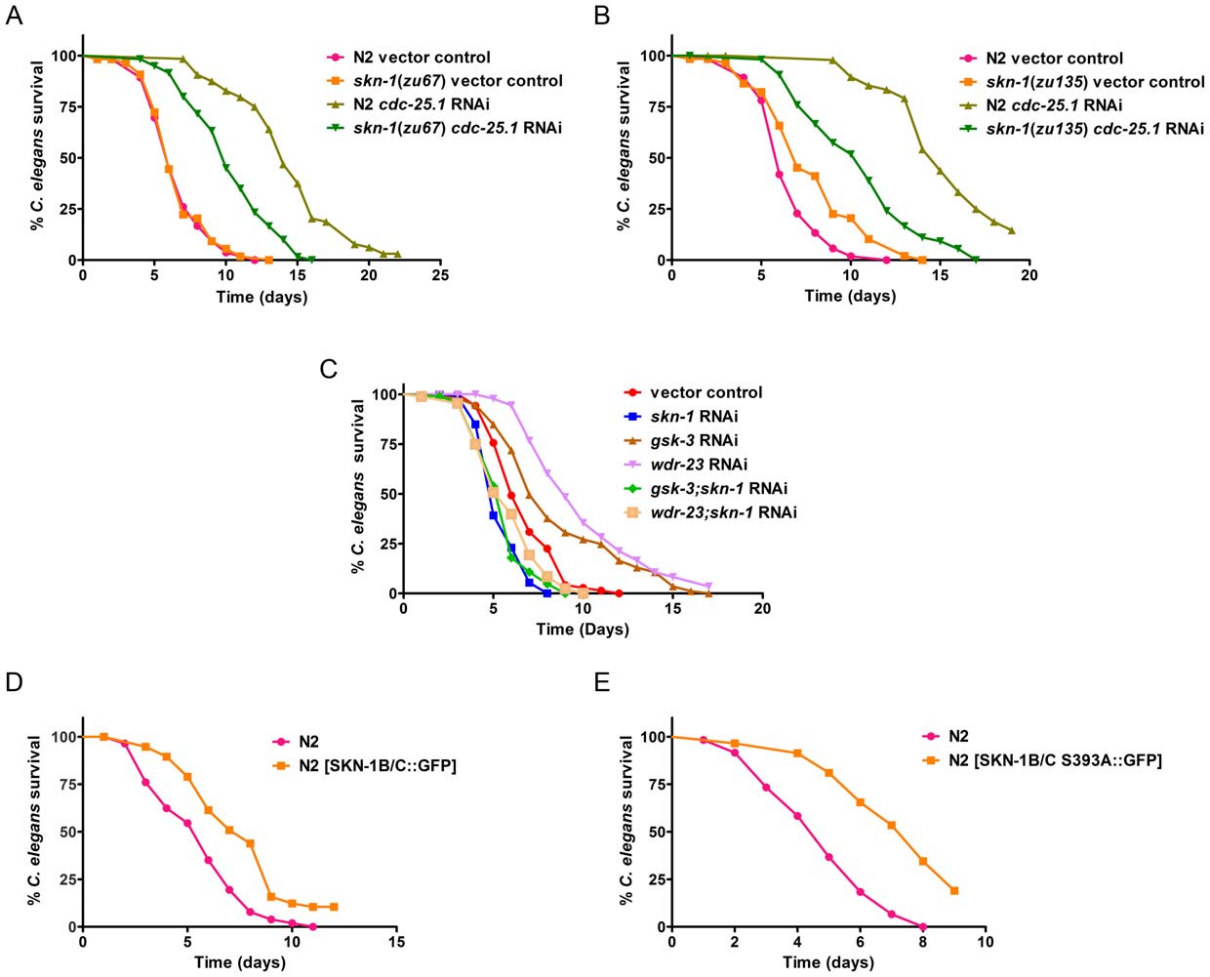
SKN-1 is required for survival of worms during infection. Animals were exposed to *E. faecalis* in all panels of this figure. (**A**) N2 and *skn-1(zu67)* worms exposed to vector control RNAi, and *cdc-25.1* RNAi, prior to pathogen exposure. (**B**) N2 and *skn-1*(*zu135*) worms exposed to vector control RNAi, and *cdc-25.1* RNAi, prior to pathogen exposure. (**C**) N2 animals exposed to vector control, *skn-1*, *gsk-3*, *wdr-23*, *gsk-3;skn-1* and *wdr-23;skn-1* RNAi. (**D**) Survival of N2 [*rol-6*(*su1006*)] and N2 [SKN-1B/C::GFP[*rol-6*(*su1006*)]] and (**E**) N2 [*rol-6*(*su1006*)] and N2 [SKN-1B/C S393A::GFP[*rol-6*(*su1006*)]]. Median survival and *P*-values are listed in Table S2A in Text S1 along with replicas of all experiments. The results of the same experiments performed on *P. aeruginosa* are shown in Table S2B in Text S1. The data are representative of experiments repeated two or more times with an n of 60 – 90 worms for each condition.

In previous investigations, loss of *skn-1* did not influence susceptibility to *P. aeruginosa* [15], [19]. One study examined loss of *skn-1* by RNAi [19]. However, in this work the animals were not exposed to RNAi until the L4 stage, at which point RNAi is likely less effective since the SKN-1 protein is produced in significant quantities during larval development [8]. We reduced *skn-1* expression using RNAi, but began the exposure at the L1 stage and continued exposure through the L4 stage. Following this experimental procedure, the animals exhibited a statistically significant and reproducible susceptibility phenotype to both *E. faecalis* (Figure 6C, Table S2A in Text S1) and *P. aeruginosa* (Table S2B in Text S1). We additionally tested the possible role in susceptibility of some individual SKN-1 targets such as *gst-4, gst-7* and *gcs-1* by reducing the expression of these genes by RNAi. A significant difference compared to control RNAi was not observed, suggesting that these are not the critical SKN-1 targets or more than one gene contributes to SKN-1 pathogen resistance (data not shown).

Another study examined loss of *skn-1* by mutation using loss of function alleles *zu67* and *zu135* [15]. Using these same strains, we also examined susceptibility to *P. aeruginosa* and additionally *E. faecalis*. In a wild-type background, there was no significant difference in susceptibility, as previously reported; in fact *zu135* was slightly more resistant (Figure 6A, 6B, Table S2 in Text S1). However, the *zu67* and *zu135* strains are sterile and do not produce viable embryos. This eliminates a major mechanism of killing, the internal hatching of the embryos during exposure to pathogen, a process called “bagging” [33], [34]. We found that RNAi of *skn-1* did not cause a severe sterility phenotype until the second generation, likely due to its maternal effect [35], so the RNAi experiments described above were not affected by this problem. To render all the strains equally sterile so that they would be directly comparable, we exposed them to *cdc-25.1* RNAi prior to pathogen exposure [36], [37]. Under these conditions, both *skn-1* mutants were significantly more susceptible compared to wild-type (Figure 6A, 6B, Table S2 in Text S1). Note that the *cdc-25.1* RNAi targets germline mitosis/meiosis, while a *skn-1* mutation affects cell division within the embryo. Preventing development of the germline is known to increase resistance [34] and likely accounts for the increase in susceptibility observed when the already sterile *skn-1* mutants are exposed to *cdc-25.1* RNAi. Shivers et al. also attempted to control for sterility by adding FUDR to their assay plates, a compound that prevents cell division [15]. We speculate that this procedure may have affected the virulence of the pathogen, as noted in previous reports [36], [38]. Since the activation of *skn-1* is very sensitive to the overall virulence of the pathogen (see Figure 2), this may have confounded the results.

To further test the role of SKN-1 on pathogen susceptibility, we used another approach. If loss of SKN-1 causes a susceptibility phenotype, constitutively active SKN-1 is predicted to increase resistance. We increased SKN-1 activity by two known means. First, we reduced expression of *gsk-3* by RNAi, which causes constitutive nuclear localization of SKN-1 and therefore, greater transcriptional activity. By phosphorylation, GSK-3 normally inhibits nuclear localization of SKN-1 [39]. Secondly, we reduced expression of *wdr-23*, which encodes a WD40 repeat protein that targets SKN-1 to an ubiquitin ligase for proteasomal degradation. Loss of WDR-23 results in increased SKN-1 protein levels and greater output of its transcriptional program [40]. Reducing the expression of both of these genes significantly increased resistance to *E. faecalis* and *P. aeruginosa* (Figure 6C, Table S2 in Text S1). As a control, the expression of *skn-1* was additionally reduced by RNAi, which abrogated the phenotypes, confirming that the increased resistance was dependent on *skn-1*. In previous work, loss of *gsk-3* or *wdr-23* both increased survival upon exposure to oxidative stress, however there was little (*wdr-23*) to no (*gsk-3*) concomitant increase in longevity when lifespan was assayed on non-pathogenic *E. coli* [39], [40]. Therefore, the significant increase in pathogen resistance observed in Figure 6A is not just a byproduct of causing a long-lived phenotype in general. To look at the effect of increased SKN-1 activity by another means, we examined two strains in which SKN-1 is over-produced because the strains carry extra copies of SKN-1::GFP. One strain carried wild-type SKN-1, fused to GFP (Figure 6D, Table S2 in Text S1), whereas the second expressed a mutant form of SKN-1, which is constitutively active (Figure 6E, Table S2 in Text S1) [8], [20]. Both strains exhibited increased resistance to both *E. faecalis* and *P. aeruginosa*. The effect was stronger utilizing the strain that produces constitutively active SKN-1 (Figure 6E, Table S2 in Text S1). Overall the data in this section demonstrates that that the level of SKN-1 activity significantly influences how long the worm survives on pathogen; less SKN-1 activity reduces resistance whereas more SKN-1 activity increases resistance.

### Epistasis Analysis of *skn-1* and *bli-3*

**Figure 7:**
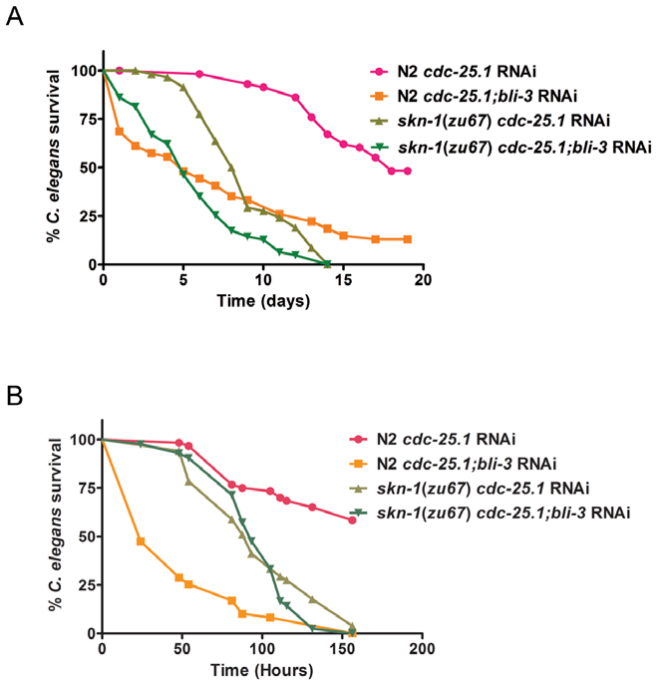
Epistasis analysis of *skn-1* and *bli-3.* Survival curves of N2 and *skn-1(zu67)* exposed to *cdc-25.1* RNAi and *cdc-25.1;bli-3* RNAi on (**A**) *E. faecalis* OG1RF and (**B**) *P. aeruginosa* PA14. Median survival and *P*-values are listed in Table S2 in Text S1 along with replicas of the experiments. The data shown is representative of experiments repeated two or more times with an n of 60 – 90 worms for each condition.

**Figure 8:**
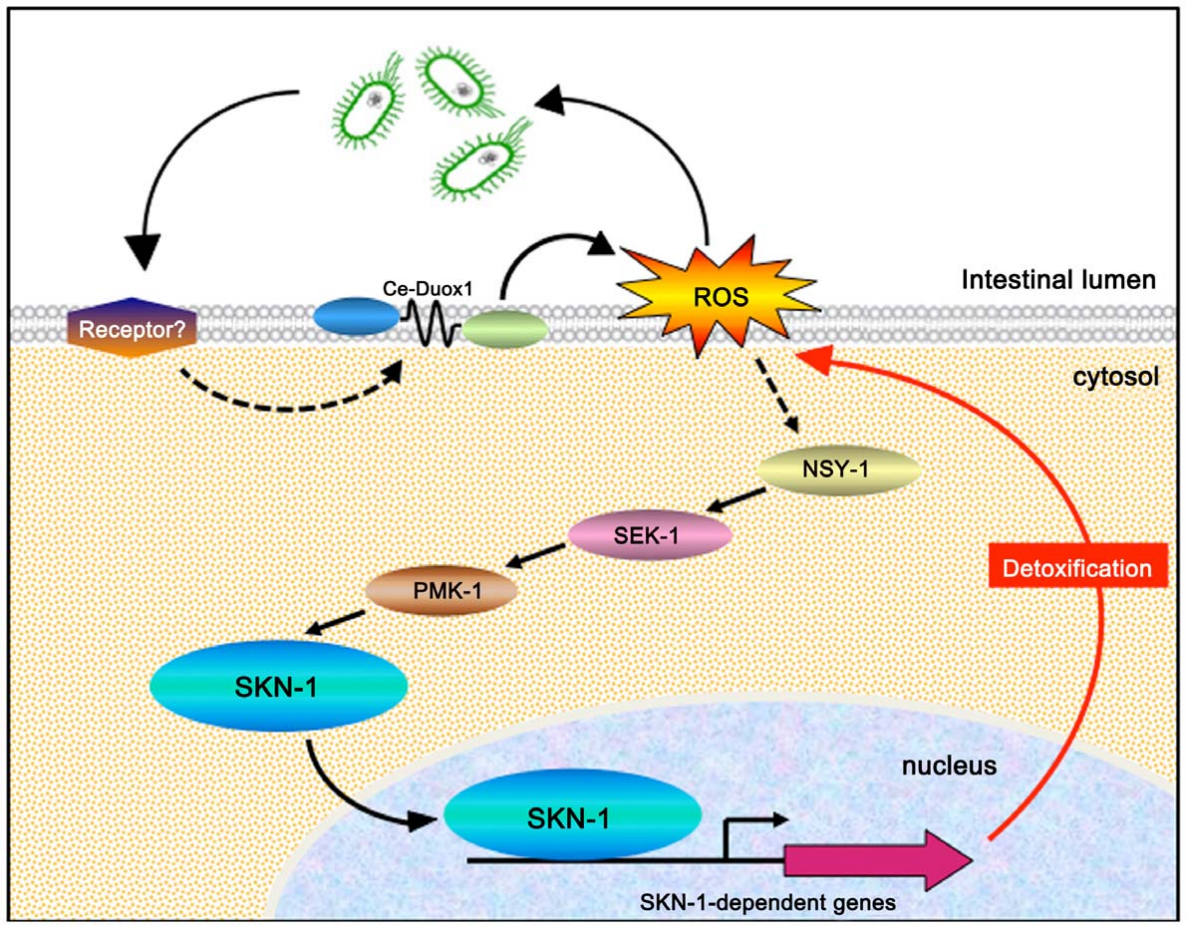
Proposed model for SKN-1 activation during infection. See the main text for a description.

In previous work, loss of *bli-3* was shown to increase susceptibility to *E. faecalis* [3]. To determine if this effect could be completely dependent on *skn-1*, phenotypic analysis of the loss of both genes on susceptibility was performed. In a *skn-1* background, loss of *bli-3* by RNAi caused an increase in susceptibility to *E. faecalis* compared to *skn-1* animals not exposed to *bli-3* RNAi (*P<*0.0001). These data suggest that *skn-1* is not completely epistatic to *bli-3,* ie not all of Ce-Duox1/BLI-3's protective effects are mediated through SKN-1 (Figure 7A, Table S2A in Text S1). However, on *P. aeruginosa*, we observed some differences (Figure 7B, Table S2B in Text S1). First, we discovered that a *bli-3* knock-down caused a profound susceptibility phenotype – much more severe than that observed on *E. faecalis* in this work and in our previous publication [3]. Loss of *skn-1* ameliorated this severe phenotype and showed the same level of susceptibility as a *skn-1* mutant plus *bli-3* RNAi (*P* = 0.3429). The data is consistent with *skn-1* being epistatic to *bli-3* on *P. aeruginosa,* despite the difference in phenotypic consequences compared to *E. faecalis*. In this case, loss of *skn-1* protects against the severe susceptibility phenotype caused by the loss of *bli-3* on *P. aeruginosa*, even though the *skn-1* mutant is still more susceptible than the wild-type strain (*P<*0.0001). Understanding why there is a difference in the *bli-3* phenotypes of animals exposed to these two different pathogens will require further investigation. Overall, the results are consistent with the model shown in Figure 8 that postulates that SKN-1 acts downstream of BLI-3. However, the experiments do not rule out the possibility that SKN-1 and BLI-3 have other roles independent of each other.

## Discussion

We have shown for the first time that the oxidative stress response transcription factor, SKN-1, plays an important role in *C. elegans* innate immunity. A model for the activation of SKN-1 is shown in Figure 8. Upon exposure to intestinal pathogens, Ce-Duox1/BLI-3 is activated by an unknown mechanism to produce extracellular ROS. In addition to possibly having direct antimicrobial properties, ROS generated by Ce-Duox1/BLI-3 (likely in the form of membrane diffusible H_2_O_2_) activates the p38 MAPK signaling cascade, which results in the phosphorylation and nuclear localization of SKN-1. SKN-1 carries out a transcriptional program to produce proteins with detoxification functions that eliminate ROS and help repair or recycle damaged molecules. Overall, we demonstrated that this response is functionally important during infection; loss of *skn-1* increased susceptibility of the worms to pathogen, whereas increasing SKN-1's activity increased resistance

Interestingly, there is evidence for Nrf-related transcription factors like SKN-1 protecting against immune-derived oxidative stress in other systems, suggesting broad conservation of this protective mechanism. A study by Jain et al. showed that the SKN-1 ortholog, Yap1 in *Saccharomyces cerevisae*, protected this microbe against Ce-Duox1/BLI-3-derived ROS produced by *C. elegans.* Specifically, it was demonstrated that a *dar* (distended anal region) phenotype caused by colonization with *S. cerevisae* was abrogated by a Yap1 mutation. The *dar* phenotype was dependent on *bli-3* [5]. Therefore it appears that Nrf-related transcription factors not only protect the host against oxidative stress during host-pathogen interactions, but also protect eukaryotic pathogens and enable their virulence. In higher animals, there is also evidence that Nrf-related transcription factors respond to immune-induced oxidative stress. For example, increasing concentrations of HOCl were shown to increase activation of Nrf2 and immune responsive genes in mouse macrophages [41]. Because Nrf transcription factors generally upregulate the production of “Phase 2” detoxification enzymes with activities such as metabolizing free radicals and conjugating xenobiotics and peroxidized lipids [10], [11], it is logical that their activity would be helpful during pathogen attack when there is often excess ROS production and cellular stress. It is therefore not surprising that one central signaling pathway, p38 MAPK signaling, has a crucial role in the *C. elegans* response to both pathogen and oxidative stress.

When considering the components of the p38 MAPK pathway that govern oxidative stress response, SEK-1 and PMK-1 have well-established functions [12]. The importance of two upstream components, NSY-1 and TIR-1 is less clear and may depend on the oxidative stressor used to assay function [12], [16], [17]. On pathogen, we observed that loss of *nsy-1* significantly reduced SKN-1 activity (Figure 4B, 4G, S2B in Text S1). The loss of *tir-1* did not cause a significant change in expression, suggesting that it is not involved (Figure 4G, S3B in Text S1). These data suggest that other components may feed into the pathway upstream of SEK-1 to activate SKN-1. Though we tested several potential candidate kinases with previously established roles in p38 MAPK signaling including MEK-1, MKK-4 and IKKε-1 [42], [43], none of them reduced SKN-1 activity (data not shown). In mammalian systems, a thioredoxin redox sensor inhibits the mammalian homolog of NSY-1, ASK-1 [44], [45]. ROS production causes the disassociation of the thioredoxin and allows an active signaling complex to form with other adaptor molecules enabling p38 MAPK signaling [46]. We are actively exploring if a thioredoxin is involved in activating NSY-1 in *C. elegans*.

In addition to SKN-1, p38 MAPK signaling in *C. elegans* was previously shown to regulate another bZIP transcription factor, ATF-7. Loss of *atf-7* enhances susceptibility to pathogen. However, rather than simply acting to induce immune activation, ATF-7 normally represses innate immune activation. Phosphorylation by PMK-1 turns ATF-7 from a repressor into an activator [15]. Since ROS released from Ce-Duox1/BLI-3 activates SKN-1 activity via p38 MAPK signaling, one could postulate that ROS also activates ATF-7. Alternatively, the signaling cascade might have some way of distinguishing between different inputs to selectively activate these transcription factors depending on the stimulus. Such a model might allow better coordination of gene expression to meet specific challenges. One gene shown to be regulated by ATF-7, *T24B8.5*, encodes a ShK-like toxin peptide which has predicted antimicrobial activity [15]. Perhaps SKN-1 coordinates a “defensive” arm of the innate immune response by regulating genes encoding enzymes involved in protecting against and repairing cellular damage, whereas ATF-7 regulates an “offensive” arm by controlling genes encoding for activities that are directly antimicrobial. A more thorough study of the genes regulated by ATF-7 and SKN-1 on pathogen would need to be carried out to investigate this hypothesis.

The precise role(s) of ROS generated by Ce-Duox1/BLI-3 in protecting the worm from infection is not yet completely understood, though this study implicates an important signaling function. On *E. faecalis*, reducing expression of *bli-3* increased the susceptibility phenotype of *skn-1*. The incomplete epistasis suggests that Ce-Duox1 has additional roles in protecting the worm from the pathogen independent of activating *skn-1.* One role could be activating other transcription factors regulated by p38 MAPK signaling such as ATF-7, as mentioned above. A potential role, independent of signaling, is in host physical barrier function. The ROS generated by Ce-Duox1/BLI-3 could be utilized by peroxidases to increase the impermeability of the ECM (extracellular matrix) in the worm intestine, analogous with how peroxidases use ROS generated by Ce-Duox1/BLI-3 to cross-link the cuticle [47]. There is some evidence for NADPH oxidases contributing to barrier function in the mosquito gut [48]. Another possibility is that ROS generated by Ce-Duox1/BLI-3, is turned into a more potent antimicrobial, as is known to happen in other systems, including the oral and respiratory mucosa of animals, in which DUOXs generate the H_2_O_2_ necessary for lactoperoxidase (LPO) to oxidize thiocyanate to create the microcidal compound hypothiocyanite [49], [50].

Our susceptibility analysis indicated that the genetic interactions between *skn-1* and *bli-3* are different on *P. aeruginosa* than on *E. faecalis*. The data in Figure 7 demonstrated that in the absence of Ce-Duox1/BLI-3, and only on *P. aeruginosa*, SKN-1 is activating a transcriptional program that is harmful to the worm, but in the presence of Ce-Duox1/BLI-3, SKN-1 is protective, as expected. On *E. faecalis*, in contrast, the presence of SKN-1 is protective in both the *bli-3* and wild-type backgrounds. Perhaps the explanation for the difference lies in the fact that *P. aeruginosa* is actively manipulating the host innate immune response through production of redox-active factors such as pyocyanin. In previous work using human respiratory epithelial cells, pyocyanin production by *P. aeruginosa* was shown to cause increased oxidative stress by potentiating the intracellular production of superoxide in the host cells. Pyocyanin, like Ce-Duox1/BLI-3 uses NADPH and molecular oxygen to create ROS, so these host and pathogen factors are potentially competing for the same substrates [27]. We postulate that intracellular superoxide production by pyocyanin, in contrast to the extracellular production of H_2_O_2_ by Ce-Duox1/BLI-3, activates a different SKN-1 transcriptional program that is very harmful to the worm. In a previous investigation, different oxidative stressors caused significant differences in SKN-1's transcriptional program, so this idea is not with out precedent [10]. This hypothesis could explain the results in Figure 7B. When Ce-Duox1/BLI-3 is present, the H_2_O_2_ produced activates SKN-1 to carry out a protective response. It may also inhibit pyocyanin activity by decreasing availability of NADPH and oxygen. Loss of *skn-1* is detrimental, but loss of *bli-3* when *skn-1* is present allows this transcription factor to be influenced by the ROS generated by the pathogen resulting in a transcriptional program that is actively harmful to the host. Testing this hypothesis will require further study.

In conclusion, we have demonstrated for the first time that the Nrf-family transcription factor SKN-1 is induced by exposure to pathogen and has a protective function during infection. We additionally showed that components of the p38 MAPK pathway, including NSY-1, SEK-1 and PMK-1, are necessary for this response. Finally, ROS produced by the dual oxidase Ce-Duox1/BLI-3 in response to pathogen was shown to trigger SKN-1 activity. Because Ce-Duox1/BLI-3 plays an important role in activating p38 MAPK signaling and SKN-1 activity, defining how this dual oxidase's activity is triggered will be an important area of future investigation.

## Materials and Methods

### Strains

*C. elegans* strains were grown and maintained as previously described [51]. The following bacterial strains were used in this study: *E. coli* OP50 [52], *E. faecalis* OG1RF [53], *P. aeruginosa* PA14 [23]. *C. elegans* strains used in this study are indicated in Table S3 in Text S1.

### RNA Isolation and Quantitative Real Time PCR (qRT-PCR) Analysis

In Figure 1A and 4G, RNA was extracted from L4 larvae exposed to *E. faecalis* OGIRF, *P. aeruginosa* PA14 and *E. coli* OP50 for 24 hours. In Figure 5I and 5J, RNAi treated *eri-1*(*mg336*) worms were exposed to *E. faecalis* OGIRF, *P. aeruginosa* PA14 and *E. coli* OP50 for 6 hours. The RNA was extracted using Trizol (Invitrogen) as indicated by the manufacturer. Samples were treated with DNase I to remove DNA contamination using the Turbo DNA free kit (Applied Biosystems) as described by the manufacturer. qRT-PCR was performed on an ABI 7500 instrument using the Power SYBR Green RNA-to-C_T_ 1 step kit (Applied Biosystems). Comparative C_T_ method was used to determine fold changes in gene expression normalized to *act-1* [20]. Primers used are listed in Table S4 in Text S1.

### RNA Interference

RNAi was induced by feeding L1 worms through L4 stage with bacteria producing dsRNA to target genes. RNAi expressing clones were obtained from the *C. elegans* library (Geneservices, UK) [54], [55]. All clones were verified by sequencing. Clones absent in the library were constructed as follows. Briefly, RNA was extracted from *C. elegans* L4 larvae using Trizol (Invitrogen) according to the manufacturer's protocol. cDNA was synthesized using SuperscriptII reverse transcriptase (Invitrogen) with oligodT and random hexamer primers. Gene specific primers were used to amplify regions of target genes, cloned into the vector pL4440 [56] and transformed into *E. coli* HT115(DE3). Constructs were verified by sequencing. Sequences of gene specific primers are listed in Table S5 in Text S1. To induce *bli-3* knockdown, the bacterial strain expressing *bli-3* RNAi was diluted in a 1∶30 ratio using the vector control. Double RNAi knockdowns were obtained by mixing bacteria expressing dsRNA to each target gene in a 1∶1 ratio. To render worms sterile prior to killing assays, larvae were exposed to *cdc-25.1* RNAi.

### Fluorescence Microscopy

To investigate the expression of *gst-4::gfp*, *gcs-1::gfp* and GST-7::GFP, worms were exposed to *E. faecalis, P. aeruginosa* and *E. coli* strains for 24 hours at 25°C and paralyzed with 1mM levamisole. Anesthetized worms were mounted on 2% agarose pads and imaged using an Olympus IX81 automated inverted microscope and Slidebook (version 5.0) software. The levels of GFP expression were scored as previously described [8], [12]. Briefly, little or no expression of GFP, expression of GFP in the anterior or posterior of the worm and expression throughout the intestine of the worm are categorically indicated by low, medium and high respectively for *gst-4*, *gcs-1* and *gst-7*. To determine the effect of hydrogen peroxide and paraquat on *gst-4::gfp* expression, worms were exposed to 5mM hydrogen peroxide or 100mM paraquat in M9 for 20 and 30 minutes respectively, then transferred to seeded NG plate to recover for 4 hours before imaging. As controls, worms were exposed to the same period of time in M9. Higher background expression was observed in the control worms using the latter procedures.

SKN-1B/C::GFP expression was analyzed by fluorescence microscopy in worms exposed to *E. faecalis* for 24 hours and *P. aeruginosa* for 6 hours. Imaging was performed using the FITC, TRITC, DAPI and YFP filter sets to exclude the signal from autofluorescence in the worms. Percentages of worms indicating the degree of nuclear localization in the intestinal cells were scored as previously described [8], [12]. Briefly, no nuclear localization, localization of SKN-1B/C::GFP in the anterior or posterior of the worm and nuclear localization of SKN-1B/C::GFP in all intestinal cells are categorically indicated by low, medium and high, respectively. All fluorescence microscopy experiments shown were independently repeated at least three times.

### Killing Assays

Killing assays were generally performed as previously described [28], [30], [57]. Briefly, for *E. faecalis* killing assays, *E. faecalis* OG1RF grown in Brain Heart Infusion (BHI) medium for 5 hours was seeded on BHI plates and incubated at 37°C for 24 hours. While for *P. aeruginosa* killing assays, *P. aeruginosa* PA14 was cultured in Luria broth (LB), seeded on slow-killing plates and incubated first for 24 hours at 37°C and then for 24 hours at 25°C. A total of 90 L4 larvae were transferred to three replica plates. Worms were scored as live and dead at various points along the time course.

### Statistical Analysis

After scoring the fluorescent micrographs, statistical differences were determined by Chi square and Fisher's exact tests using GraphPad Prism version 5.0 (GraphPad Software, San Diego, CA). Each experimental condition was compared pairwise to the control condition. *P*-values of <0.05 were considered to be statistically significant. Statistically significant differences are indicated in the figures with asterisks next to the experimental condition. Kaplan-Meier log rank analysis was used to compare survival curves pairwise and to calculate the median survival. *P*-values <0.05 were considered to be statistically significant.

## Supporting Information

Text S1 Supporting figures, tables and references.
(DOC)
