# Supplementary material for: Ce-Duox1/BLI-3 Generated Reactive Oxygen Species Trigger Protective SKN-1 Activity via p38 MAPK Signaling during Infection in C. elegans
Source: PLoS Pathog. 2011 Dec 22;7(12):e1002453. doi: 10.1371/journal.ppat.1002453 (PMC3245310; doi:10.1371/journal.ppat.1002453)
Supplement: Text S1 — Supporting figures, tables and references. (DOC) [file ppat.1002453.s001.doc]

**TEXT S1**

**Figures**

**

**

**Figure S1. GST-7::GFP is Produced in Response to Pathogens.** Representative Normaski and fluorescent views of worms expressing GST-7::GFP exposed to (**A**) *E. faecalis* OG1RF, (**B**) *P. aeruginsoa* PA14, (**C**) *E. coli* OP50 for 18 hours. (**D**) The level of GST-7::GFP expression was scored and the percentage of worms in each category is indicated along with the number of worms observed (n). *E. faecalis* OG1RF and *P. aeruginosa* PA14 caused higher levels of GST-7::GFP expression (*P* < 0.0001) compared to *E. coli* OP50.

**
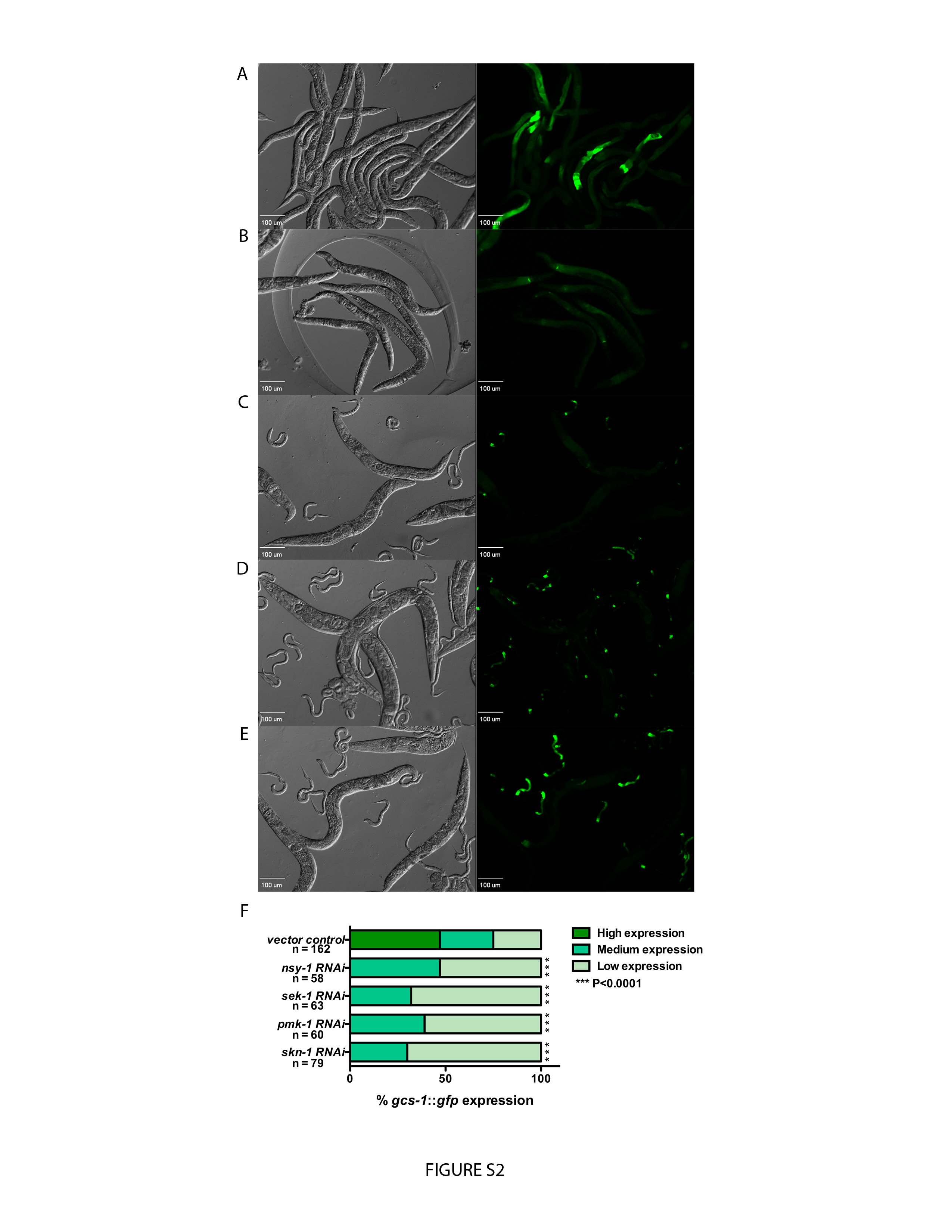
**

**Figure S2. Activation of SKN-1 is Dependent on p38-MAPK Pathway in Response to Pathogens.** Worms expressing *gcs-1::gfp* were exposed to (**A**) control, (**B**) *nsy-1*, (**C**) *sek-1*, (**D**) *pmk-1*, and (**E**) *skn-1* RNAi prior to feeding on *P. aeruginosa* for 18 hours. (**F**) The level of *gcs-1::gfp* expression was scored and the percentage of worms in each category is indicated along with the number of worms observed (n). Significantly higher levels of expression of *gcs-1::gfp* were observed in worms exposed to the control RNAi (*P* < 0.0001) compared to *nsy-1*, *sek-1*, *pmk-1* and *skn-1* RNAi.





**Figure S3. Activation of SKN-1 is Not Dependent on TIR-1 in Response to Pathogens.** Worms expressing *gst-4::gfp* were exposed to (**A**) control, (**B**) *tir-1* RNAi prior to feeding on *E. faecalis* for 18 hours. (**C**) The level of *gst-4::gfp* expression was scored and the percentage of worms in each category is indicated along with the number of worms observed (n). No significant difference was observed in worms exposed to the control RNAi compared to *tir-1* RNAi (*P* = 0.2205).

**Tables**

**Table S1. Percentage Expression of *gst-4::gfp* Exposed to *E. coli* OP50 following RNAi Knock-down of the Following Genes.**

| **RNAi clone** | **Number of worms** | **Percentage expression of *gst-4::gfp*** | | |
| --- | --- | --- | --- | --- |
|  |  | **High** | **Medium** | **Low** |
| control | 149 | 6 | 24 | 70 |
| *bli-3* | 43 | 14 | 23 | 63 |
| *nsy-1* | 71 | 6 | 32 | 62 |
| *pmk-1* | 73 | 21 | 10 | 69 |
| *sek-1* | 60 | 2 | 3 | 95 |
| *skn-1* | 37 | 5 | 0 | 95 |
| *tir-1* | 51 | 16 | 8 | 76 |

**Table S2A. Median Survival and *P*-values of *E. faecalis* OG1RF Pathogenicity Assays**

| **Figure number** | **Experiment number** | **Strain** | **Median survival (days)** | ***P*-value*** |
| --- | --- | --- | --- | --- |
| 6A | 1 | N2 | 6 | C |
|  |  | *skn-1*(*zu67*) | 6 | NS |
|  |  | N2, *cdc-25.1* RNAi | 14 | C |
|  |  | *skn-1*(*zu67*), *cdc-25.1* RNAi | 10 | <0.0001 |
|  | 2 | N2 | 6 | C |
|  |  | *skn-1*(*zu67*) | 6 | NS |
|  |  | N2, *cdc-25.1* RNAi | 14 |  |
|  |  | *skn-1*(*zu67*), *cdc-25.1* RNAi | 8 | <0.0001 |
| 6B | 1 | N2 | 6 | C |
|  |  | *skn-1*(*zu135*) | 7 | =0.0012 |
|  |  | N2, *cdc-25.1* RNAi | 15 |  |
|  |  | *skn-1*(*zu135*), *cdc-25.1* RNAi | 11 | <0.0001 |
|  | 2 | N2 | 6 | C |
|  |  | *skn-1*(*zu135*) | 7 | =0.0016 |
|  |  | N2, *cdc-25.1* RNAi | 14 |  |
|  |  | *skn-1*(*zu135*), *cdc-25.1* RNAi | 10 | <0.0001 |
| 6C | 1 | vector control | 6 | C |
|  |  | *skn-1* RNAi | 5 | <0.0001 |
|  |  | *gsk-3* RNAi | 7 | <0.0001 |
|  |  | *wdr-23* RNAi | 9 | <0.0001 |
|  |  | *gsk-3* skn-1 RNAi | 6 | <0.0001 |
|  |  | *wdr-23* skn-1 RNAi | 6 | =0.0029 |
|  | 2 | vector control | 6 | C |
|  |  | *skn-1* RNAi | 5 | <0.0001 |
|  |  | *gsk-3* RNAi | 7 | <0.0001 |
|  |  | *wdr-23* RNAi | 9 | <0.0001 |
|  |  | *gsk-3*;*skn-1* RNAi | 6 | <0.0001 |
|  |  | *wdr-23;skn-1* RNAi | 6 | <0.0001 |
| 6D | 1 | [*rol-6*] | 6 | C |
|  |  | [SKN-1B/C::GFP[rol*-6*]] | 8 | <0.0001 |
|  | 2 | [*rol-6*] | 6 | C |
|  |  | [SKN-1B/C::GFP[*rol-6*]] | 7 | <0.001 |
|  | 3 | [*rol-6*] | 6 | C |
|  |  | [SKN-1B/C::GFP[*rol-6*]] | 7 | <0.01 |
| 6E | 1 | [*rol-6*] | 5 | C |
|  |  | [SKN-1B/C S393A::GFP[*rol-6*]] | 8 | <0.0001 |
|  | 2 | [*rol-6*] | 6 | C |
|  |  | [SKN-1B/C S393A::GFP[*rol-6*]] | 7 | <0.001 |
|  | 3 | [*rol-6*] | 6 | C |
|  |  | [SKN-1B/C S393A::GFP[*rol-6*]] | 7 | <0.001 |
| 7A | 1 | N2, *cdc-25.1* RNAi | 18 | C |
|  |  | *skn-1*(*zu67*), *cdc-25.1* RNAi | 8.5 | <0.0001 |
|  |  | N2, *cdc-25.1;bli-3* RNAi | 5 | <0.0001 |
|  |  | *skn-1*(*zu67*), *cdc-25.1;bli-3* RNAi | 5 | <0.0001 |
|  | 2 | N2, *cdc-25.1*;RNAi | 12 | C |
|  |  | *skn-1*(*zu67*), *cdc-25.1* RNAi | 8 | <0.0001 |
|  |  | N2, *cdc-25.1;bli-3* RNAi | 6 | <0.0001 |
|  |  | *skn-1*(*zu67*), *cdc-25.1;bli-3* RNAi | 6 | <0.0001 |

*****Corresponds to a pair-wise comparison of the survival curves, the tested condition(s) to the control for each experiment. Some experiments have two controls and shading indicates how the comparisons were grouped. The control is indicated by a “C.” NS stands for “not significant.”

**Table S2B. Median Survival and *P*-values of *P. aeruginosa* PA14 Pathogenicity Assays**

| **Figure number** | **Experiment number** | **Strain** | **Median survival (hours)** | ***P*-value*** |
| --- | --- | --- | --- | --- |
| 6A** | 1 | N2 | 74.5 | C |
|  |  | *skn-1(zu67)* | 70.57 | NS |
|  |  | N2, *cdc-25.1* RNAi | 148.5 | C |
|  |  | *skn-1*(*zu67*), *cdc-25.1* RNAi | 102.5 | <0.0001 |
|  | 2 | N2 | 76 | C |
|  |  | *skn-1(zu67)* | 72.5 | NS |
|  |  | N2, *cdc-25.1* RNAi | 117.5 | C |
|  |  | *skn-1*(*zu67*), *cdc-25.1* RNAi | 92.5 | <0.0001 |
| 6B** | 1 | N2 | 74.5 | C |
|  |  | *skn-1*(*zu135*) | 70.5 | =0.0012 |
|  |  | N2, *cdc-25.1* RNAi | 148.5 | C |
|  |  | *skn-1*(*zu135*), *cdc-25.1* RNAi | 102.5 | <0.0001 |
|  | 2 | N2 | 76 | C |
|  |  | *skn-1*(*zu135*) | 68.5 | =0.0016 |
|  |  | N2, *cdc-25.1* RNAi | 117.5 | C |
|  |  | *skn-1*(*zu135*), *cdc-25.1* RNAi | 92.5 | <0.0001 |
| 6C** | 1 | vector control | 65 | C |
|  |  | *skn-1* RNAi | 64 | <0.0001 |
|  |  | *gsk-3* RNAi | 89 | <0.0001 |
|  |  | *wdr-23* RNAi | 73 | <0.0001 |
|  |  | *gsk-3*;*skn-1* RNAi | 48 | <0.0001 |
|  |  | *wdr-23*;*skn-1* RNAi | 48 | =0.0029 |
|  | 2 | vector control | 67 | C |
|  |  | *skn-1* RNAi | 63 | <0.0001 |
|  |  | *gsk-3* RNAi | 90 | <0.0001 |
|  |  | *wdr-23* RNAi | 75 | <0.0001 |
|  |  | *gsk-3*;*skn-1* RNAi | 49 | <0.0001 |
|  |  | *wdr-23*;*skn-1* RNAi | 49 | <0.0001 |
| 6D,E** | 1 | [*rol-6*] | 1 | C |
|  |  | [SKN-1B/C::GFP[*rol-6*]] | 2.5 | <0.01 |
|  |  | [SKN-1B/C S393A::GFP[*rol-6*]] | 3 | <0.01 |
|  | 2 | [*rol-6*] | 1 | C |
|  |  | [SKN-1B/C::GFP[*rol-6*]] | 2 | <0.01 |
|  |  | [SKN-1B/C S393A::GFP[*rol-6*]] | 3 | <0.01 |
| 7B | 1 | N2, *cdc-25.1* RNAi | 171.25 | C |
|  |  | *skn-1*(*zu67*), *cdc-25.1* RNAi | 94.25 | <0.0001 |
|  |  | N2, *cdc-25.1;bli-3* RNAi | 24 | <0.0001 |
|  |  | *skn-1*(*zu67*), *cdc-25; bli-3* RNAi | 94.25 | <0.0001 |
|  | 2 | N2, *cdc-25.1* RNAi | 171.25 | C |
|  |  | *skn-1*(*zu67*), *cdc-25.1* RNAi | 93.25 | <0.0001 |
|  |  | N2, *cdc-25.1;bli-3* RNAi | 24 | <0.0001 |
|  |  | *skn-1*(*zu67*), *cdc-25.1;bli-3* RNAi | 93.25 | <0.0001 |

*Corresponds to a pair-wise comparison of the survival curves, the tested condition(s) to the control for each experiment. Some experiments have two controls and shading indicates how the comparisons were grouped. The control is indicated by a “C.” NS stands for “not significant.” **Figure number corresponds to figure of *E. faecalis* killing assay.

**Table S3. Strains Used in this Study**

**Description Reference**

Wild type Bristol strain N2

*skn-1*(*zu67*) IV/nT1[*unc-?(n754) let-?*](IV;V) [1]

*skn-1*(*zu135*) IV/nT1[*unc-?(n754) let-?*](IV;V) [1]

CL2166: dvIs19[pAF15(*gst-4*::*gfp*::NLS)] [2]

LD002: N2 Is007[SKN-1B/C::GFP;*rol-6*(*su1006*)] [3]

LD1171: N2 Is003[*gcs-1::gfp;rol-6*(*su1006*)] [3]

LD1052: N2 Ex701[*gst-7::gfp;rol-6*(*su1006*)] [4]

N2 dgEx3[*rol-6*(*su1006*)] this work

LD1252: N2 Ex020[SKN-1B/C S393A::GFP;*rol-6*(*su1006*)] [4]

*eri-1*(*mg336*) [5]

*tir-1*(*qd4*) III [6]

*nsy-1*(*ag3*) II [7]

*sek-1*(*ag1*) X [7]

*pmk-1*(*km25*) IV [8]

**Table S4. List of Primers Used for qRT-PCR**

**Primer Sequence**

*act-1* QRT F ACCATGTACCCAGGAATTGC

*act-1* QRT R TGGAAGGTGGAGAGGGAAG

*bli-3* QRT F AGAGCTTATCACGCATTCCG

*bli-3* QRT R CAAATAACACGATGGGACCAAC

*gcs-1* QRT F TGTTGATGTGGATACTCGGTG

*gcs-1* QRT R TGTATGCAGGATGAGATTGTACG

*gst-4* QRT F CGTTTTCTATGGAAGTGACGC

*gst-4* QRT R TCAGCCCAAGTCAATGAGTC

*gst-5* QRT F AGTCACTCAGGAACAATGGC

*gst-5* QRT R GAGCCAAGAAACGAGCAATC

*gst-7* QRT F GGACAAGACTTCGAGGACAAC

*gst-7* QRT R AACTGACGAGCCAAGTAACG

*gst-10* QRT F AAGAGATTGTGCAGACTGGAG

*gst-10* QRT R AGAACATGTCGAGGAAGGTTG

*skn-1* QRT F GACGTCAATTTATGGAGTGTCG

*skn-1* QRT R GAAGATGTTTTGTCGTGATCCG

**Table S5. List of Primers Used for Construction of RNAi Clones**

**Primer Sequence**

*pmk-1* F CATGCCATGGAGTACGGGCAGCATGTATAGC

*pmk-1* R CGGGGTACCTCTCCTCATCTTCCTCTTCGTCAG

*sek-1* F CATGCCATGGCGAGTCCGAAGAGATTGAGATTGC

*sek-1* R CGGGGTACCCTCTCCATCAATTCTCTCTGGTGG

* Underlined sequences denotes restriction endonuclease recognition site

**References**

1. Bowerman B, Eaton BA, Priess JR (1992) skn-1, a maternally expressed gene required to specify the fate of ventral blastomeres in the early C. elegans embryo. Cell 68: 1061-1075.

2. Link CD, Johnson CJ (2002) Reporter transgenes for study of oxidant stress in Caenorhabditis elegans. Methods Enzymol 353: 497-505.

3. An JH, Blackwell TK (2003) SKN-1 links C. elegans mesendodermal specification to a conserved oxidative stress response. Genes Dev 17: 1882-1893.

4. Tullet JM, Hertweck M, An JH, Baker J, Hwang JY, et al. (2008) Direct inhibition of the longevity-promoting factor SKN-1 by insulin-like signaling in C. elegans. Cell 132: 1025-1038.

5. Kennedy S, Wang D, Ruvkun G (2004) A conserved siRNA-degrading RNase negatively regulates RNA interference in C. elegans. Nature 427: 645-649.

6. Shivers RP, Pagano DJ, Kooistra T, Richardson CE, Reddy KC, et al. (2010) Phosphorylation of the conserved transcription factor ATF-7 by PMK-1 p38 MAPK regulates innate immunity in Caenorhabditis elegans. PLoS Genet 6: e1000892.

7. Kim DH, Feinbaum R, Alloing G, Emerson FE, Garsin DA, et al. (2002) A conserved p38 MAP kinase pathway in *Caenorhabditis elegans* innate immunity. Science 297: 623-626.

8. Kim DH, Liberati NT, Mizuno T, Inoue H, Hisamoto N, et al. (2004) Integration of Caenorhabditis elegans MAPK pathways mediating immunity and stress resistance by MEK-1 MAPK kinase and VHP-1 MAPK phosphatase. Proc Natl Acad Sci U S A 101: 10990-10994.
